# Bio-Based Hydroxypropyl Methylcellulose Reinforced Water Glass/Silica Sol Hybrid Gel Foam with Synergistic Flame-Retardant and Enhanced Fireproof Performance Under Laboratory Screening Conditions for Forest Fire Barriers

**DOI:** 10.3390/ma19122434

**Published:** 2026-06-07

**Authors:** Pengfei Wang, Zhiming Bai, Ruoxin Cong, Hongyu Yang

**Affiliations:** 1 Tianjin Key Laboratory of Fire Safety Technology, Tianjin Fire Science and Technology Research Institute of MEM, Tianjin 300381, China; 2 School of Resources and Safety Engineering, University of Science and Technology Beijing, Beijing 100083, China; crx41927294@163.com; 3 College of Materials Science and Engineering, Chongqing University, Chongqing 400044, China

**Keywords:** bio-based hybrid gel foam, forest fire barriers, synergistic enhancement, water retention, flame retardancy

## Abstract

**Highlights:**

- A composite of water glass/HPMC/silica sol (SGF-HPMC-SOL) was constructed.
- A stable organic–inorganic hybrid network enhances the material’s properties.
- Its flame-retardant performance is superior to that of traditional gels.

**Abstract:**

To meet the requirements of forest fire prevention, a water glass-based composite gel foam was developed by introducing hydroxypropyl methylcellulose (HPMC) and nanosilica sol into a sodium silicate/sodium bicarbonate matrix. The resulting water glass/HPMC/silica sol ternary system (SGF-HPMC-SOL) was designed to improve water retention, foam stability, substrate adhesion, and fire-barrier durability. The results indicate that HPMC and silica sol contributed to network reinforcement through hydrogen bonding, polymer-chain entanglement, nanoscale filling, and possible interfacial condensation. The optimized SGF-HPMC-SOL retained 20.4% of its initial mass after heating at 100 °C for 5 h, compared with 4.65% for SGF and 9.54% for SGF-HPMC; reached a carbonization time of 164 s under direct-flame exposure, versus 100 s for SGF and 137 s for SGF-HPMC; and maintained a residual mass of 76% at 800 °C in TGA, compared with 58.3% for SGF and 55.1% for SGF-HPMC. These improvements were associated with the formation of a denser silica-rich protective layer after combustion, which delayed heat transfer to the wood substrate. Under the adopted direct-flame screening conditions, SGF-HPMC-SOL exhibited enhanced flame-retardant performance compared with the reference gel foams, indicating its potential for enhanced flame-retardant performance under laboratory screening conditions for forest fire prevention.

## 1. Introduction

Wildfires have become increasingly frequent and destructive under the combined effects of climate warming, prolonged drought, and fuel accumulation [1,2,3,4]. Therefore, materials that can be rapidly applied to combustible vegetation or wooden substrates to form temporary fire barriers are important for proactive forest fire prevention. However, practical fire-barrier materials used in open forest environments must meet several coupled requirements, including water retention, substrate adhesion, foam stability, structural integrity after drying, and sustained thermal shielding during flame exposure. These requirements define the main research gap addressed in this study.

Current wildland fire chemical systems are generally classified into long-term retardants, foam suppressants, and water-enhancing gels [5]. Long-term retardants, such as phosphate-based systems, can remain effective as long as the chemical residue is retained on vegetation. However, they are often designed for large-scale aerial or landscape applications rather than forming a localized gel barrier on wood or vegetation surfaces. Foam suppressants and water-enhancing gels provide cooling and wetting effects, but their protection depends strongly on water retention. Once the applied water evaporates, their fire resistance decreases substantially. Water-enhancing gels typically provide a longer water retention window than Class A foams, but they may still lose effectiveness after dehydration under high heat and wind [5]. Therefore, next-generation forest fire-barrier gels should not only retain water and adhere to substrates but also preserve a protective inorganic-rich structure after water loss.

Water glass-based gels are attractive candidates for forest fire barriers because sodium silicate is inexpensive, environmentally benign, and capable of forming an inorganic Si–O–Si network after gelation. Nevertheless, conventional water glass gels usually suffer from poor water retention, shrinkage-induced cracking, pulverization after dehydration, limited adhesion to inclined or vertical biomass surfaces, poor foam stability, and inadequate long-term flame-retardant durability. Previous studies have demonstrated that composite gel strategies can improve the rheological, mechanical, oxidation-resistant, and flame-retardant properties of gel systems [6,7,8,9,10,11,12]. For example, graphene-silicone polymer composite gels [8], soluble carboxymethyl starch/tannic acid-modified inorganic gels [9], solid-waste-filled composite gels [10,11], and thermosensitive konjac glucomannan/expandable graphite gels [12] have been developed for fire prevention or extinguishment applications. However, many of these systems were designed for coal mine fire prevention or focused on improving one dominant property, such as mechanical strength, oxidation resistance, or inert filling. They do not fully address the coupled requirements of water retention, foam stabilization, substrate adhesion, and post-combustion barrier formation in open forest fire prevention scenarios.

Recent studies have shown that silica-containing hydrogel systems can improve fire protection by combining water retention, surface adhesion, and condensed-phase thermal shielding. Yu et al. developed a sprayable viscoelastic fire-retardant fluid composed of biopolymers and colloidal silica to improve retention on wildfire-prone vegetation [13]. Dong et al. reported cellulosic polymer/colloidal silica water-enhancing gels that transformed into thermally insulating silica aerogel coatings under flame exposure and protected substrates for several minutes after dehydration [5]. Xu et al. prepared a silica hydrogel-based forest fire extinguishing agent; after treatment, the total heat release and total smoke release of pine were reduced by 51.05% and 72.86%, respectively [14]. These studies support the use of silica-containing hydrogel systems for wildfire protection. Unlike previous silica-hydrogel systems that use preformed colloidal silica particles as physical crosslinkers and form bulk hydrogels, our work uses water glass as a polymerizable inorganic precursor to form a foam structure, where the water glass matrix itself provides the flame-retardant barrier after dehydration without requiring added polyphosphate retardants. However, few studies have integrated a flexible bio-based polymer and an active nanosilica phase into a water glass foam system to simultaneously improve water retention, foam stability, adhesion to biomass substrates, and silica-rich residue formation after combustion.

HPMC and silica sol were selected in this study because they provide complementary functions. HPMC contains abundant hydroxyl and ether groups, which can improve water binding, viscosity, film formation, and interfacial adhesion through hydrogen bonding and polymer-chain entanglement. Silica sol contains nanoscale SiO_2_ particles with surface silanol groups, which can fill microvoids, reinforce the inorganic framework, and contribute to a silica-rich protective residue during combustion. The simultaneous incorporation of HPMC and silica sol into a water glass matrix is therefore expected to form a denser hybrid network through hydrogen bonding, physical entanglement, nanoscale filling, and possible interfacial condensation. This design differs from single-component modification strategies because it aims to overcome several coupled failure modes of water glass gels rather than improving only one isolated property.

Based on this rationale, the central hypothesis of this study is that a water glass/HPMC/silica sol hybrid gel foam can exhibit higher water retention, foam stability, stackability, adhesion, thermal stability, and flame-retardant durability than unmodified water glass gel foam and water glass/HPMC binary gel foam. To test this hypothesis, a sodium silicate/sodium bicarbonate water glass matrix was modified with HPMC and alkaline silica sol, and sodium dodecyl sulfate was used as the foaming agent. The gelation behavior, water retention, foaming performance, microscopic foam morphology, viscosity, stackability, adhesion, thermal stability, combustion resistance, and post-combustion structure were evaluated. FTIR, TGA, SEM, and XRD analyses were used to clarify the relationship between the suggested organic–inorganic hybrid structure and the fire-protective mechanism.

## 2. Materials and Methods

### 2.1. Materials

Powdered instant sodium silicate (Na_2_O·3SiO_2_, Shandong Keyuan Biochemical Co., Ltd., Heze, China, AR, powder) was used as the water glass precursor. Sodium bicarbonate (NaHCO_3_, Sinopharm Chemical Reagent Co., Ltd., Shanghai, China, AR, powder) was used as the gelation promoter. Hydroxypropyl methylcellulose (HPMC, Fuqiang Chemical, Shijiazhuang, China, powder) was used as the organic polymer modifier. Alkaline silica sol (mSiO_2_·nH_2_O, Guangzhou Fufei Chemical Technology Co., Ltd., Guangzhou, China, solution) was used as the nanosilica source; its particle size was 14–30 nm, solid content was 30 wt%, and pH was 9–10.

### 2.2. Preparation of Composite Gels

All gel preparation experiments were conducted under the same laboratory ambient conditions of 25 ± 2 °C and 50 ± 5% relative humidity. In this study, three gel samples were prepared for comparative analysis, with the preparation procedures as follows.

(1) Water glass gel (labeled as SG)

Weigh 0.6 g of sodium silicate (Na_2_O·3SiO_2_) and dissolve it in 10 mL of deionized water. Sonicate the mixture for 30 min to ensure complete dissolution, resulting in Solution A. Separately, weigh 0.6 g of sodium bicarbonate (NaHCO_3_) and dissolve it in 10 mL of deionized water. Stir magnetically for 3 min until fully dissolved to obtain Solution B. Subsequently, pour Solution B into Solution A, stir continuously for 20 s, and allow the mixture to stand, yielding the base water glass gel.

(2) Water glass/HPMC composite gel (labeled as SG-HPMC)

Weigh 0.6 g of sodium silicate (Na_2_O·3SiO_2_) and 0.16 g of hydroxypropyl methylcellulose (HPMC), and add them together into 10 mL of deionized water. Sonicate the mixture for 30 min, supplemented with magnetic stirring for 2 min, to completely dissolve the solids, resulting in Solution A. Prepare Solution B by dissolving 0.6 g of sodium bicarbonate in 10 mL of deionized water using the method described above. Finally, pour Solution B into Solution A, stir for 20 s, and allow the mixture to stand and solidify, obtaining the water glass/HPMC composite gel.

(3) Water glass/HPMC/silica sol composite gel (labeled as SG-HPMC-SOL)

Solution A is prepared using the same method as for the SG-HPMC sample. For Solution B, add 3.33 mL of alkaline silica sol into a solution of 0.6 g sodium bicarbonate in 10 mL deionized water, and stir for 20 s to achieve a homogeneous mixture. Finally, pour the mixed Solution B into Solution A, stir for 20 s, and allow the mixture to stand, forming the ternary water glass/HPMC/silica sol composite gel.

### 2.3. Preparation of Composite Gel Foam

All foam preparation experiments were conducted under the same laboratory ambient conditions of 25 ± 2 °C and 50 ± 5% relative humidity. Foaming was performed using a high-speed stirrer at 20,000 r/min for 1 min.

(1) Water glass gel foam (labeled as SGF)

Weigh 3.0 g of sodium silicate and dissolve it in 50 mL of deionized water. Sonicate the mixture for 30 min to prepare Solution A. Separately, weigh 3.0 g of sodium bicarbonate and 1.0 g of sodium dodecyl sulfate (SDS), and dissolve them together in 50 mL of deionized water. Stir magnetically for 5 min until complete dissolution to obtain Solution B. Solution A and Solution B were transferred into a high-speed stirrer and foamed at 20,000 r/min for 1 min. The mixture was then poured out to obtain the water glass gel foam.

(2) Water glass/HPMC gel foam (labeled as SGF-HPMC)

Weigh 3.0 g of sodium silicate and 0.8 g of HPMC, and add them together into 50 mL of deionized water. Sonicate the mixture for 30 min and stir for 2 min to dissolve, serving as Solution A. Prepare Solution B containing sodium bicarbonate and SDS according to the method used for SGF preparation. Solution A and Solution B were transferred into the high-speed stirrer and foamed at 20,000 r/min for 1 min. The mixture was then poured out to obtain the water glass/HPMC composite gel foam.

(3) Water glass/HPMC/silica sol gel foam (labeled as SGF-HPMC-SOL)

Solution A is prepared in the same manner as for SGF-HPMC. Solution B is also prepared for SGF. Additionally, measure 16.67 mL of alkaline silica sol as Component C. Finally, Solution A, Solution B, and Component C were placed together into the high-speed stirrer and foamed at 20,000 r/min for 1 min to prepare the ternary water glass/HPMC/silica sol composite gel foam.

### 2.4. Performance Tests of Composite Gels

To systematically evaluate the comprehensive performance of the prepared silicon-based curing foam materials, the gelation time and water retention of the gel systems, as well as the foaming performance, stackability, adhesion, and flame retardancy of the foams were tested in this study. Furthermore, various instrumental characterization methods were employed to analyze the viscosity, microscopic morphology, thermal stability, functional group structure, post-combustion morphology, and crystalline composition of the foams. To improve the rigor and reproducibility of the experimental evaluation, unless otherwise specified, sample preparation and room-temperature tests were conducted under laboratory ambient conditions of 25 ± 2 °C and 50 ± 5% relative humidity. For quantitative measurements (gelation time, water retention, foaming performance, stackability, adhesion, combustion test, and viscosity), each experiment was repeated three times using independently prepared batches of each formulation. The results are presented as mean ± standard deviation (SD), and the error bars in the figures represent the SD of three independent replicates.

(1) Determination of gelation time

The gelation time of the samples was measured using the inclined test tube method. This visual flow-stop method has been widely used as a simple and practical approach for estimating the gelation time of silica gel and hydrogel systems under static conditions [15]. Briefly, a test tube containing the uniformly mixed gel slurry was placed under controlled laboratory conditions of 25 ± 2 °C and 50 ± 5% relative humidity and tilted at a 45° angle at fixed intervals. The gelation endpoint was defined as the time at which no visible flow was observed within 30 s after tilting.

It should be noted that the inclined test tube method provides a practical macroscopic indication of the sol–gel transition, but its endpoint may require visual judgment. Therefore, the viscosity evolution of different gel foams was further measured using a digital viscometer as a complementary quantitative rheological indicator. The viscosity–time curves were used to compare the evolution of flow resistance and network formation among SGF, SGF-HPMC, and SGF-HPMC-SOL.

(2) Water retention test

A formed gel sample of a certain initial mass (W_0_) was placed in a blast drying oven at 100 °C. The sample was removed at one-hour intervals, and its current mass (Wᵢ) was measured using an analytical balance until the mass stabilized. The water retention rate (WR) was calculated using the following formula.

WR (%) = (Wᵢ/W_0_) × 100% (1)

The test at 100 °C was designed as an accelerated dehydration experiment to compare the intrinsic water retention capacity of different gel formulations under identical thermal conditions.

(3) Foaming performance test

To screen the optimal foaming system, four types of surfactants (at 1% concentration) were selected for foaming performance research, including anionic (sodium dodecyl sulfate, SDS), cationic (cetyltrimethylammonium bromide, CTAB), zwitterionic (dodecyl dimethyl betaine, BS-12), and nonionic (polyoxyethylene sorbitan monooleate, Tween 80). Each surfactant was mixed separately with three types of gel base solutions and stirred for 1 min using agitation equipment. The foaming volume was measured immediately, and the foam half-life, that is, the time required for the foam volume to decay to half after standing, was recorded to comprehensively evaluate its foaming capacity and foam stability.

(4) Stackability test

A polytetrafluoroethylene ring with an inner diameter of 5 cm and a height of 2 cm was placed on a pine wood board. The ring was filled with freshly prepared gel foam and leveled. After initial solidification, the mold was carefully removed to form a cylindrical foam body. The remaining height was measured after standing for 10 min to evaluate the self-supporting ability and shape retention of the gel foam under unconfined conditions.

(5) Adhesion test

Freshly prepared gel foam was evenly applied onto a vertically placed pine wood board to form a coating with a thickness of 1 cm, and the initial mass (M_0_) was recorded. After curing for 30 min, the gel debris detached from the board surface was collected and weighed (M_l_). The adhesion rate (AR) was calculated using the following formula. This gravimetric method provides a semi-quantitative evaluation of adhesion.

AR (%) = [(M_0_ − M_l_)/M_0_] × 100% (2)

(6) Combustion test

A 10 g gel foam sample was uniformly applied onto the surface of a pine wood board (8 cm × 8 cm), confined to a 5 cm × 5 cm area, forming a coating with an approximate thickness of 1 cm. The combustion resistance was evaluated using a butane blow torch, with the nozzle center positioned 5 cm away from the gel foam surface. Timing started when the flame contacted the sample and continued until distinct carbonization marks appeared on the wooden substrate. The recorded duration was defined as the carbonization time, which was used as a comparative indicator of the persistent fire resistance of the gel foam.

This blow-torch exposure test was used as an accelerated comparative screening method to evaluate the protective duration of different gel foam formulations under direct flame impingement. Similar time-to-char tests have been used for water-enhancing fire-protective gels. For example, Dong et al. applied hydrogel coatings onto plywood substrates, exposed them to a hand torch stabilized approximately 5 cm above the gel surface, and recorded the time required for the wood substrate to visually char [5]. Their method was also described as a variation of the “Evaluation of Wildland Fire Chemicals Standard Test Procedures” adapted from ASTM E1321-1997(02) [5]. To reduce experimental variability, the sample mass, coating area, coating thickness, torch-to-sample distance, substrate type, and carbonization criterion were kept constant for all samples. Each combustion test was repeated three times, and the results are reported as mean ± SD.

(7) Viscosity test

The viscosity of the samples was measured using a digital viscometer (NDJ-8S, Shanghai Fangrui Instrument Co., Ltd., Shanghai, China) at room temperature (25 ± 2 °C). According to the rotor/speed selection table provided by the instrument manufacturer, an appropriate rotor and rotational speed were selected for each sample based on its estimated viscosity range. The viscosity value was recorded after the displayed reading stabilized.

(8) Microstructural morphology analysis

The morphology of different foams at room temperature and their changes over time were observed using a Jingrui Optical HD-4800W stereo microscope (Zhongshan Jingrui Optical Technology Co., Ltd., Zhongshan, China). Fresh foam was placed in a petri dish with a diameter of 10 cm and a height of 1 cm. Images were captured at t = 0 min and t = 30 min, and image analysis software (Kingsoft Antivirus V16, version 16.2026.2.1.052815.1335) was used to calculate the bubble size distribution, investigating the influence of the gel system and fly ash aggregate on foam size and stability.

(9) Thermogravimetric analysis

Thermogravimetric tests were performed on three sets of samples using a TA TGA 550 thermal analyzer from the United States to analyze the inhibitory effect of the silicon-based solidified foam on wood powder combustion and its own thermal stability (TA Instruments, New Castle, DE, USA). The experiment measured the real-time mass change of the samples with temperature under programmed heating conditions, with a temperature range set from 30 °C to 800 °C, a heating rate of 10 °C/min, and an air flow rate of 50 mL/min.

(10) Fourier transform infrared (FTIR) spectroscopy analysis

The functional group structures of the dried gel foam samples were analyzed using a Nicolet iS20 FTIR spectrometer (Thermo Fisher Scientific, Waltham, MA, USA) over a wavenumber range of 4000–400 cm^−1^.

(11) Scanning electron microscopy (SEM) analysis

The microscopic morphology of the post-combustion silicon-based solidified foam was observed using a ZEISS Gemini SEM 300 field emission environmental scanning electron microscope (Carl Zeiss Microscopy GmbH, Oberkochen, Germany). Prior to testing, the samples were sputter-coated with gold for 150 s. The accelerating voltage was set between 5–15 kV, the magnification range was 12× to 1,000,000×, and the Inlens secondary electron detector was used.

(12) X-ray diffraction (XRD) analysis

The crystalline composition of the surface of the post-combustion silicon-based solidified foam was analyzed using a Rigaku Ultima IV X-ray diffractometer (Rigaku Corporation, Tokyo, Japan). The measurement was performed with a copper target, a scanning angle range of 5–90°, and a scanning speed of 2°/min.

(13) Forest fire barrier test

In this study, the forest fire barrier test was conducted using pine needles to simulate the forest combustible surface layer under conditions of an ambient temperature of 23.5 ± 3.5 °C and a wind speed of 15.5 ± 3.5 km/h. In the test, 65 kg of pine needles with a moisture content of 6.36 ± 0.41% were evenly spread across a 3 m × 6 m horizontal area. Half of the pine needle area was sprayed with the material (SGF-HPMC-SOL) at a flow rate of 50 L/min for one minute to create a fire barrier, while the other half remained untreated. After the material was sprayed on one half of the area, the untreated pine needles were immediately ignited from the side farthest from the treated area. Under the above experimental conditions, two replicate tests were conducted. During the experiment, a camera was used to record the fire spread process.

## 3. Results and Discussion

### 3.1. Analysis of the Gelation Mechanism of Composite Gels

**Figure 1:**
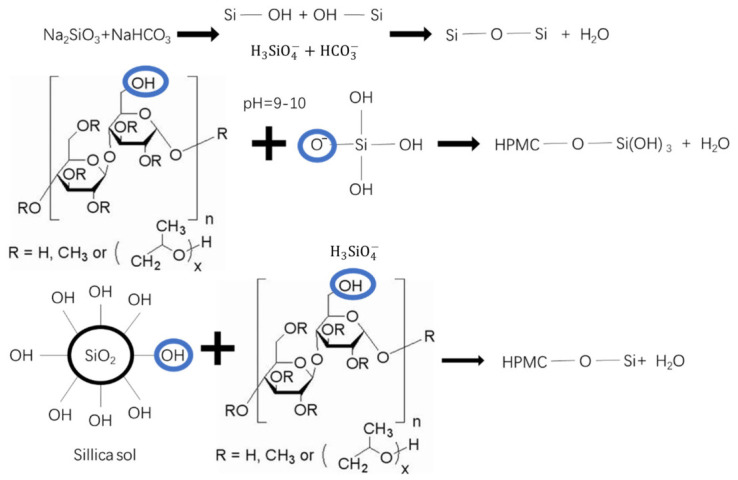
Gel mechanism of inorganic–organic composite gel.

Figure 1 shows the gel mechanism of inorganic–organic composite gel. The gelation mechanism of the SGF–HPMC–silica sol system is mainly attributed to the combined effects of physical entanglement, hydrogen bonding, and possible inorganic–organic interactions. During gel formation, HPMC chains undergo hydration and expansion in the aqueous phase, leading to the formation of a three-dimensional network through chain entanglement and intermolecular hydrogen bonding [16,17].

Meanwhile, the silica sol particles are uniformly dispersed within the polymer matrix. Under alkaline conditions, the surface silanol groups (Si–OH) of silica sol may interact with the hydroxyl groups of HPMC. FTIR spectra show changes in characteristic absorption bands, suggesting the presence of interactions between the organic polymer and inorganic phase.

These observations indicate that silica sol is not only physically embedded within the HPMC network but may also participate in the formation of a hybrid structure. In particular, the interaction between Si–OH groups and hydroxyl groups of HPMC may lead to the possible formation of Si–O–C linkages, thereby enhancing the structural stability of the gel network [18].

Overall, the gel network in this system is considered to be a hybrid structure formed through physical entanglement, hydrogen bonding, and possible inorganic–organic interactions, which together contribute to the improved stability and performance of the composite gel.

### 3.2. Determination of Composite Gel Formulations

#### 3.2.1. The Influence of the Concentration of Each Component on the Gelation Time

**Figure 2:**
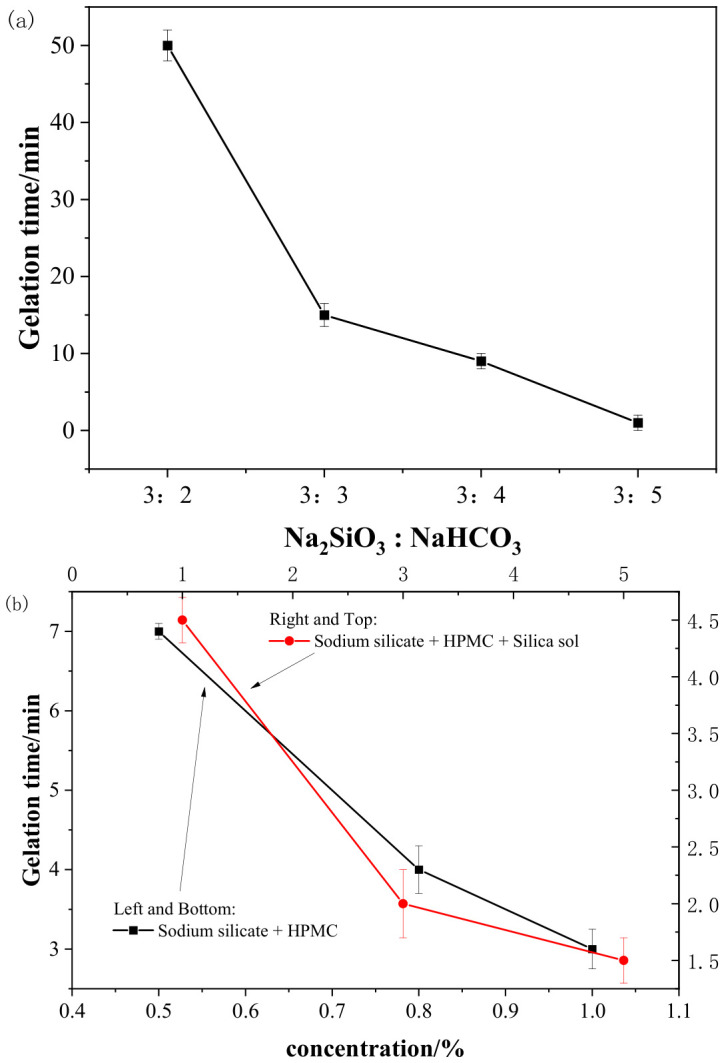
Influencing factors of gelation time of composite gel (**a**) Mass ratio of NaHCO_3_/Na_2_O·3SiO_2_ (**b**) concentration of HPMC and silica sol.

As shown in Figure 2a, the gelation time gradually shortens with the increase in the mass ratio of base material to gelling promoter, and the gelation state improves accordingly. As the mass ratio of sodium silicate to sodium bicarbonate increases, the gelation time of the pure water glass gel decreases. Considering the presence of foam in the system, if the gelation time is too short, the foaming agent may not have sufficient time to foam adequately; if too long, the formed foam may gradually collapse. Therefore, based on Figure 2a, the mass ratio of Na_2_SiO_3_ to NaHCO_3_ = 3:3 (concentrations both at 3%) was selected, giving a gelation time of 15 min for subsequent experiments. As shown in Figure 2b, the addition of hydroxypropyl methylcellulose (HPMC) reduces gelation time, decreasing further with increasing HPMC concentration. Incorporation of silica sol further shortens gelation time, with higher content leading to a shorter gelation time. Each experiment was independently repeated three times; the error bars in Figure 2 represent the standard deviation (SD) of three replicates. The optimal formulation was selected by balancing gelation time and foam stability: too short gelation impedes foaming, whereas too long gelation allows foam collapse. Thus, 3% Na_2_SiO_3_, 3% NaHCO_3_, 0.8% HPMC, and 5% silica sol provide a practical compromise.

#### 3.2.2. The Influence of Hydroxypropyl Methylcellulose and Silica Sol on Water Retention Capacity

The water retention performance of the composite gel is a key determinant of its flame-retardant durability. This study significantly enhances the water retention of the water glass gel by incorporating hydroxypropyl methylcellulose (HPMC) and silica sol, with the enhancement mechanism mainly stemming from the synergistic effects of hydrogen bonding, physical adsorption, nanoscale filling, and possible interfacial condensation.

(1) Impact of HPMC incorporation on water retention

The HPMC molecular chains are rich in hydrophilic functional groups such as hydroxyl and ether groups, and their water retention mechanism is primarily manifested in two aspects. Firstly, these functional groups themselves can serve as binding sites, capturing a large number of water molecules through hydrogen bonding, thereby converting free water into bound water. Secondly, extensive intermolecular hydrogen bonds can form between the hydroxyl groups on the HPMC chains and the silanol groups (Si-OH) in the inorganic siloxane network. This interaction achieves physical cross-linking between the organic polymer chains and the inorganic skeleton, which not only enhances the overall stability of the gel network and reduces pathways for water diffusion but also, through the extension of HPMC chains in water, creates a hydrophilic polymer phase that permeates the system, thereby enabling uniform distribution and fixation of water.

**Figure 3:**
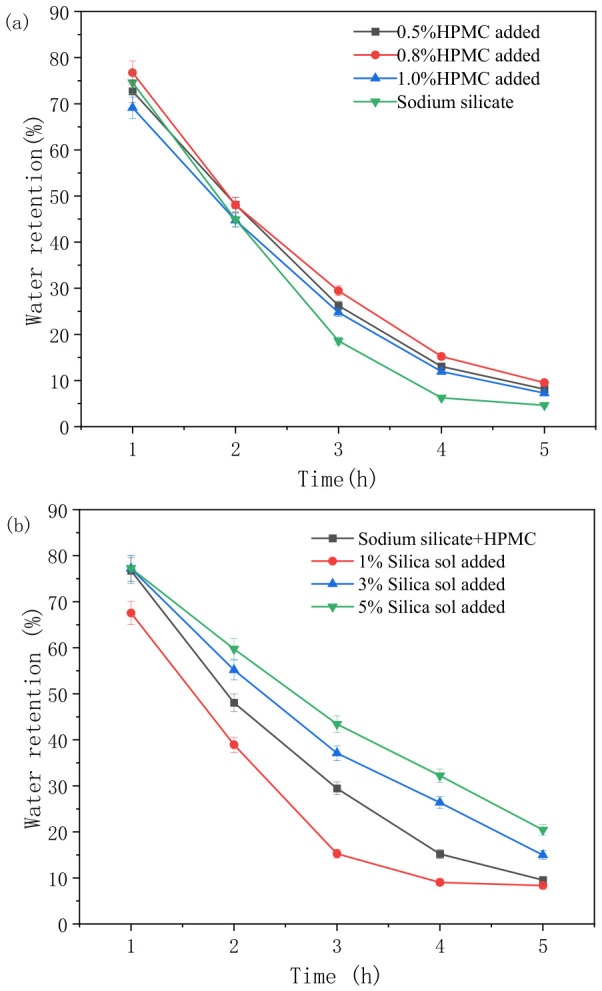
Curve of water retention rate of composite gel with time (**a**) Different HPMC concentrations (**b**) Different concentrations of silica sol.

However, as shown in Figure 3a, there is an optimal value for the amount of HPMC added. When the concentration is too high, water retention decreases. This phenomenon may result from several contributing factors. First, local agglomeration and deterioration of network homogeneity mean that excess HPMC molecular chains may form local aggregates due to van der Waals forces and hydrogen bonding, potentially disrupting network uniformity. Second, increased system viscosity can trap bubbles during stirring, which may facilitate water migration. Finally, an imbalance in structural strength means that the mechanically weaker HPMC network may be more prone to collapse under heat, contributing to accelerated water loss.

(2) Enhancement of water retention by the incorporation of silica sol

As shown in Figure 3b, the addition of silica sol improves the water retention of the gel, as it further reinforces the water-retaining structure of the gel at the nanoscale. The nano-SiO_2_ particles possess a large specific surface area and abundant surface silanol groups. These particles effectively fill the microscopic pores of the gel, making the matrix more compact and physically hindering water migration. In addition, under alkaline conditions, the silanol groups on the surface of silica sol may interact with the hydroxyl groups on HPMC chains through hydrogen bonding and possible condensation-related interactions. These interfacial interactions, combined with the physical filling effect of nano-SiO_2_ particles, likely contribute to increased water retention by strengthening the hybrid network and reducing pathways for water migration.

#### 3.2.3. Preferred Foaming Agent

**Figure 4:**
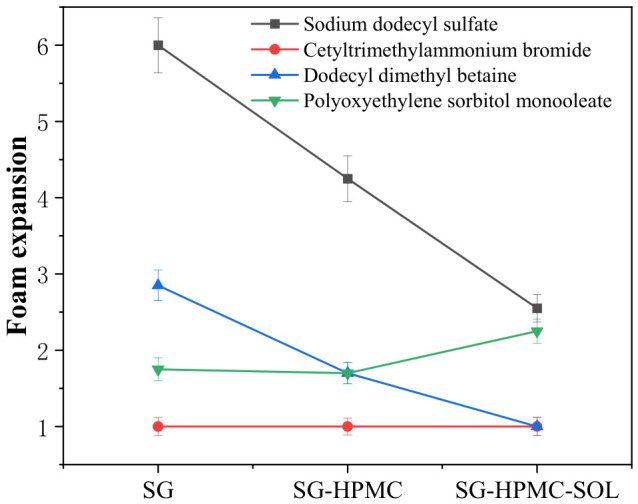
Comparison of foaming properties of different surfactants.

The foaming agent critically influences the microstructure and flame-retardant performance of gel foam materials. To identify a suitable foaming agent for the sodium silicate/sodium bicarbonate solidification system, four types of surfactants were systematically evaluated in terms of electrical compatibility and molecular structure efficiency. The results indicate that under the weakly alkaline conditions, anionic surfactants exhibit the best compatibility and foaming efficiency due to the electrostatic repulsion between their hydrophilic head groups and the dominant anions in the system. In contrast, cationic surfactants fail due to electrostatic complexation, while zwitterionic and nonionic surfactants are limited by the steric hindrance of their molecular head groups and interfacial arrangement efficiency, respectively. Detailed results are shown in Figure 4. The foaming agents investigated include anionic sodium dodecyl sulfate, cationic cetyltrimethylammonium bromide, zwitterionic dodecyl dimethyl betaine, and nonionic polyoxyethylene sorbitan monooleate.

As shown in Figure 4, the anionic surfactant sodium dodecyl sulfate (SDS) exhibits the most superior overall foaming performance, with its foaming expansion ratio significantly outperforming the other three. This phenomenon can be reasonably explained at the molecular interaction mechanism level.

In the weakly alkaline (pH ≈ 9–10) sodium silicate system, the dodecyl sulfate anions (C_12_H_25_OSO_3_^−^) generated by SDS dissociation experience electrostatic repulsion from the dominant silicate anions (e.g., H_3_SiO_4_^−^) and OH^−^ ions, which helps prevent ineffective adsorption and flocculation with the inorganic components. This likely allows SDS molecules to diffuse toward the gas–liquid interface efficiently. The linear dodecyl tail may contribute to interfacial film formation, potentially enhancing foam stability. Consequently, the high compatibility of SDS with this gel system and its interfacial activity are expected to support its superior foaming expansion and stability. In contrast, CTAB exhibited no significant foaming due to irreversible electrostatic complexation with silicate ions, generating insoluble precipitates and eliminating surface activity.

Although dodecyl dimethyl betaine (BS-12) exhibits a negative charge under alkaline conditions, making it electrically compatible with the system, its bulky betaine head group introduces significant steric hindrance. This effect severely impedes the tight packing of molecules at the interface, resulting in structural defects in the formed liquid film and insufficient strength, thereby leading to poor foam stability and a limited foaming expansion ratio.

Polyoxyethylene sorbitan monooleate (Tween 80) relies on hydrogen bonding for hydration and does not engage in electrostatic interactions with ions. However, its highly branched hydrophobic tail (oleic acid chain) and bulky polyoxyethylene hydrophilic head collectively result in low arrangement efficiency and packing density at the gas–liquid interface. Although its foaming capacity is acceptable, the strength and stability of the foam liquid film it forms cannot match those of the structurally regular SDS.

In summary, the foaming performance of a surfactant is the result of the combined effects of its molecular structure and the specific chemical environment of the base material. In the sodium silicate-based composite gel system of this study, sodium dodecyl sulfate (SDS) has been established as the most suitable foaming agent due to its optimal electrical compatibility, regular molecular configuration, and efficient interfacial adsorption capability. Subsequent research will be conducted based on this finding.

### 3.3. Microscopic Morphology Analysis of Silicon-Based Curing Foam

Foam systems are thermodynamically unstable, and their decay primarily occurs through three mechanisms: liquid film drainage, gas diffusion (Ostwald ripening), and film rupture. The introduction of functional components can significantly intervene in these processes and extend foam lifetime.

**Figure 5:**
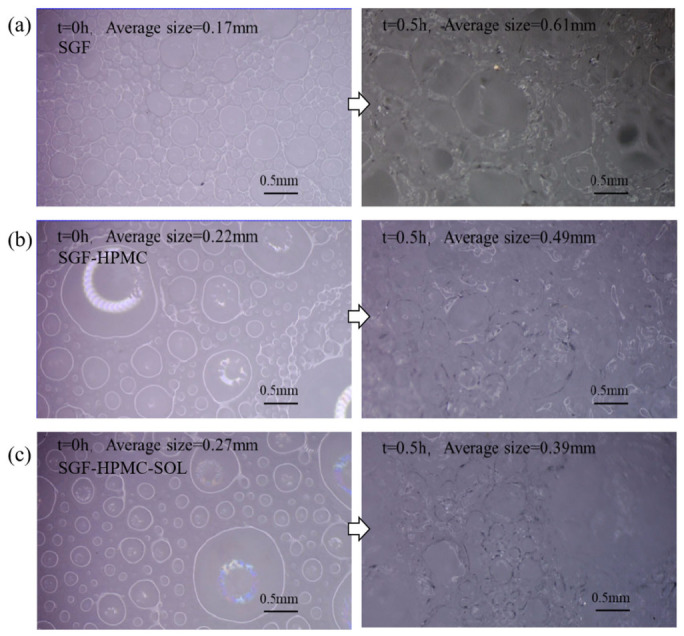
Microscopic morphologies of different composite foams (**a**) SGF, (**b**) SGF-HPMC, (**c**) SGF-HPMC-SOL.

Figure 5 shows the microscopic morphologies of the three composite foam samples, which allow for a quantitative assessment of their stabilizing effects. Although the SGF-HPMC-SOL sample had a slightly larger initial bubble size (0.27 mm), it demonstrated the lowest bubble size growth rate after 30 min (44.4%). This value is significantly lower than that of the pure SGF (258.8%) and the SGF-HPMC foam (122.7%), indicating that the composite gelling system effectively suppresses gas diffusion from small to large bubbles, thereby enhancing dimensional stability. The stabilization mechanism of HPMC primarily involves viscosity enhancement and liquid film reinforcement, which collectively retard foam decay. First, dissolved HPMC significantly increases the viscosity of the liquid phase, slowing drainage and reducing liquid flow in the Plateau borders. Second, HPMC molecules may adsorb at the gas–liquid interface, forming an elastic and viscous adsorbed layer that potentially hinders bubble coalescence. The observed trends are consistent with Ostwald ripening and liquid film drainage phenomena.

### 3.4. Viscosity Analysis of Silicon-Based Curing Foam

**Figure 6:**
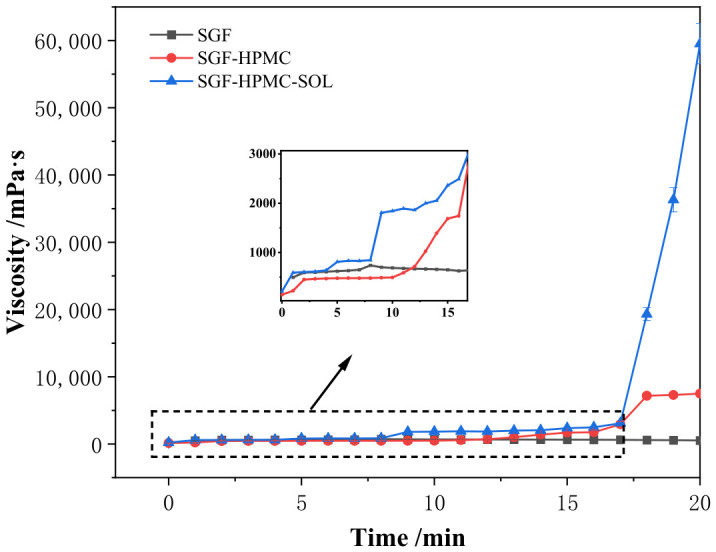
Viscosity curves of composite gel foams under different compositions.

As shown in Figure 6, the water glass gel foam incorporating hydroxypropyl methylcellulose and silica sol exhibits the highest viscosity, while the pure water glass gel foam shows the lowest viscosity. In the solution of sodium bicarbonate and sodium silicate, although the introduction of sodium dodecyl sulfate (SDS) can generate foam, the system viscosity remains the lowest. This is because a single surfactant can only form a weak monomolecular film at the gas–liquid interface, resulting in fragile bubble walls prone to drainage and leading to poor foam stability and low viscosity. When hydroxypropyl methylcellulose (HPMC) is pre-added, the viscosity of the foaming system increases significantly. This is because HPMC, on the one hand, substantially enhances the bulk viscosity of the solution, effectively slowing down the drainage process of the foam liquid films; on the other hand, its polymer chains adsorb at the interface to form a viscoelastic protective film, reinforcing the bubble structure and collectively hindering bubble coalescence and deformation, thereby exhibiting higher flow resistance. When both HPMC and alkaline silica sol are present in the system, the foam viscosity reaches its maximum. The silica sol nanoparticles not only act as physical fillers, but their surface hydroxyl groups may interact with HPMC hydroxyl groups through possible condensation-related interactions, forming a reinforced hybrid network within the bubble liquid films. This structure helps lock the bubbles, enhancing the mechanical stability of the foam and resulting in highly viscous and stable semi-solid behavior.

### 3.5. Analysis of Stackability and Adhesion of Silicon-Based Curing Foams

**Figure 7:**
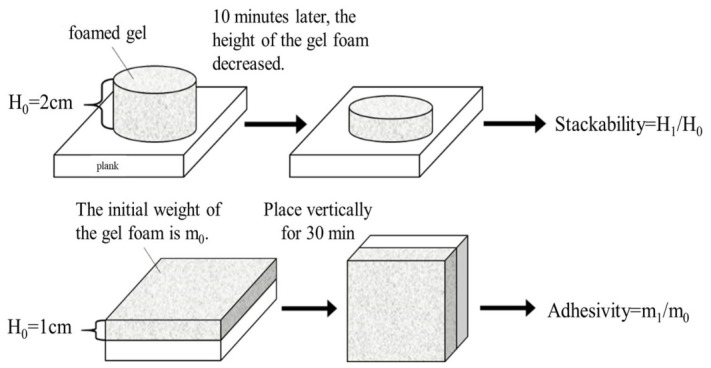
Flow chart of the stackability and adhesiveness tests.

**Figure 8:**
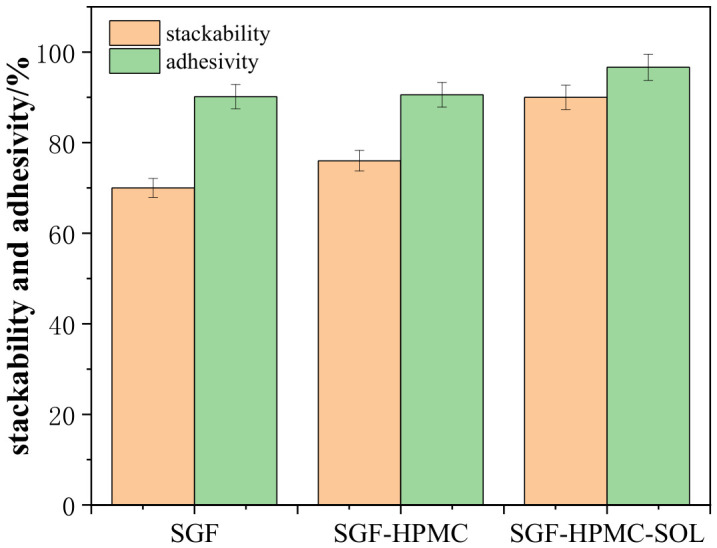
Stackability (H_1_/H_0_) and adhesion (m_1_/m_0_) of the three foam gel formulations. H_1_/H_0_ is the ratio of remaining height after 10 min to initial height; m_1_/m_0_ is the ratio of adhered mass after 30 min to initial mass. Data are presented as mean ± SD (*n* = 3). The error bars are based on three independent replicate measurements.

As shown in Figure 7 and Figure 8, the stacking performance and adhesion of silicon-based curing foams exhibit significant differences, with the performance ranking consistently being as follows: ternary composite system (SGF-HPMC-SOL) > binary composite system (SGF-HPMC) > base system (SGF). This order clearly demonstrates the synergistic effect of organic polymers and nano-inorganic particles in enhancing the mechanical properties and interfacial adhesion of the gel.

The stacking performance (H_1_/H_0_) of the gel foam reflects the ability of its cured network structure to resist deformation under its own weight. For the base system, its brittle pure siloxane network has low mechanical strength and is prone to brittle fracture and structural collapse under gravity, resulting in the poorest stacking performance. After introducing HPMC to form a binary composite system, the flexible polymer long chains and the rigid inorganic skeleton interpenetrate, forming a typical interpenetrating network structure. This structure can effectively dissipate stress through viscoelastic behaviors such as molecular chain extension and retraction, significantly improving the toughness of the gel, thereby enabling better shape retention under unconfined conditions. When silica sol is further introduced to form the ternary composite system, the silanol groups on the surface of the nano-SiO_2_ particles may interact with the hydroxyl groups on the HPMC chains, forming a reinforced hybrid network. This network helps improve the stiffness and structural stability of the foam, enabling it to better resist creep deformation caused by gravity. The adhesion (m_1_/m_0_) was semi-qualitatively assessed; the ternary system exhibited stronger attachment, likely due to hydrogen bonding and other physical interactions at the interface.

In the binary composite system, the polar functional groups on HPMC molecular chains, such as hydroxyl and ether groups, may interact with cellulose and hemicellulose on the pine wood surface through hydrogen bonding, potentially enhancing interfacial adhesion. Simultaneously, physical cross-linking by HPMC may also improve the bulk cohesive strength of the gel, making it less susceptible to internal failure. In the ternary composite system, the combination of HPMC and silica sol likely reinforces the hybrid network, which may contribute to improved cohesion of the gel and enhanced interfacial interactions with the wood substrate. These effects collectively suggest a trend of increased adhesion without asserting specific chemical bond formation or absolute adhesion values.

In summary, HPMC appears to enhance the toughness, cohesive strength, and interfacial affinity of the gel through polymer-chain entanglement and hydrogen bonding. The addition of silica sol further reinforces the network through nanoscale filling and possible organic–inorganic interfacial interactions, collectively constructing a more stable hybrid structure with improved mechanical stability and substrate adhesion.

### 3.6. Thermogravimetric Analysis of Silicon-Based Curing Foams

**Figure 9:**
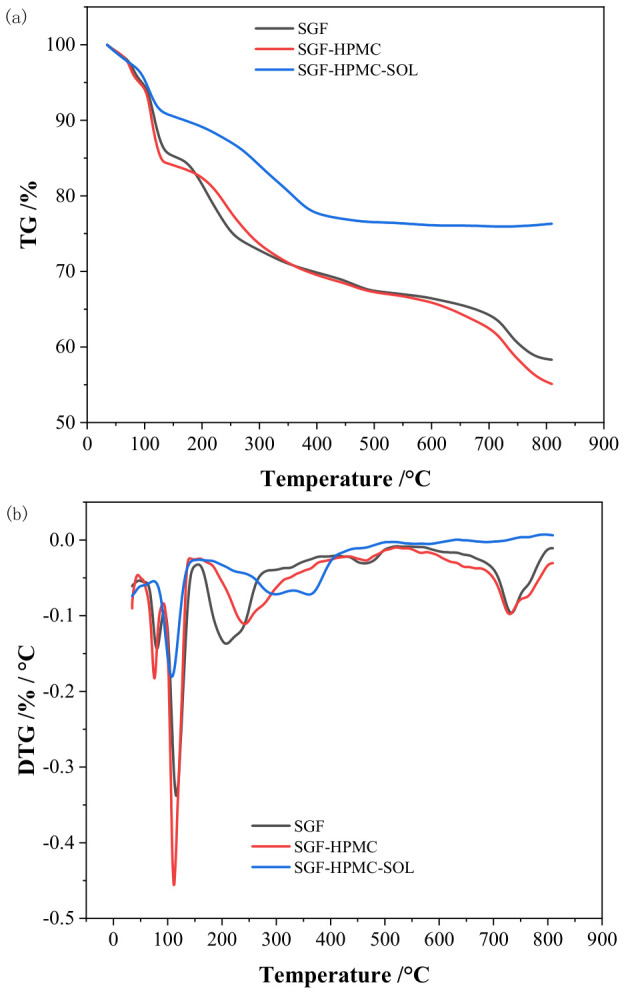
Thermal stability analysis of composite gel foam (**a**) TG curve (**b**) DTG curve.

Figure 9 shows the TG and DTG curves of SGF, SGF-HPMC, and SGF-HPMC-SOL. To provide a quantitative comparison of the thermal behavior, the characteristic DTG peak temperatures (Tmax) corresponding to the maximum mass-loss rates in each stage were analyzed. SGF-HPMC-SOL exhibits slower mass loss at 100–400 °C, consistent with enhanced water retention and a stabilized network structure. At 800 °C, the residual mass of SGF-HPMC-SOL was 76%, indicating the presence of a stable silica-rich inorganic framework.

In the low-temperature region below 150 °C, the mass loss is mainly associated with the evaporation of free water and weakly bound water. The shift of the main low-temperature DTG peak to 107 °C in SGF-HPMC-SOL suggests that the ternary hybrid network modifies the binding state and migration pathway of water molecules. HPMC hydroxyl groups and the silanol-rich silica sol may jointly strengthen water–network interactions, contributing to improved water retention behavior.

In the medium-temperature region, the broader coupled peaks at 299–360 °C for SGF-HPMC-SOL indicate that the thermal degradation of the organic component is delayed and more gradual, likely due to the reinforced organic–inorganic hybrid network. Nanoscale silica filling and stronger interfacial interactions may restrict polymer-chain mobility and promote stepwise carbonization within a silica-rich framework.

In the high-temperature region, no distinct DTG peak near 730 °C is observed for SGF-HPMC-SOL, suggesting improved stability of the residual inorganic network. Overall, the TGA/DTG results suggest that incorporation of HPMC and silica sol modifies both the dehydration behavior and the thermal degradation pathway (as reflected in Tmax values), which is consistent with improved structural integrity and long-lasting flame-retardant performance of the ternary composite gel foam.

### 3.7. Fourier Transform Infrared Spectroscopy Analysis of the Dried Silicon-Based Curing Gel

**Figure 10:**
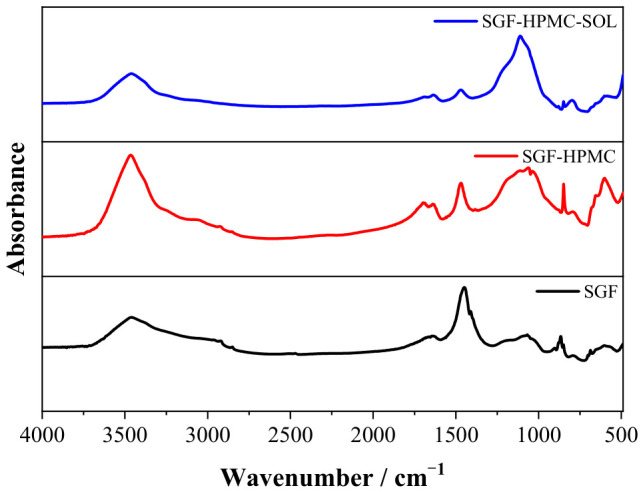
Fourier transform infrared spectra of water glass and its composite gels.

To further clarify the structural evolution of the composite gel system, FTIR spectra were collected for the dried SGF, SGF-HPMC, and SGF-HPMC-SOL samples. Figure 10 shows the fourier transform infrared spectra of water glass and its composite gels. The analysis focused on the characteristic bands related to hydroxyl groups, carbonate species, and silicate networks, including the bands near 3500, 1440, 1100, and 850 cm^−1^. These bands provide information on hydrogen bonding, silicate condensation, and organic–inorganic interactions in the composite gel system.

The broad absorption band near 3500 cm^−1^ is assigned to the stretching vibration of O–H groups, mainly originating from adsorbed water, silanol groups (Si–OH), and hydroxyl groups in HPMC [19]. Compared with SGF, the SGF-HPMC sample shows a stronger and more pronounced O–H absorption band, which can be attributed to the introduction of abundant hydroxyl groups from HPMC and the formation of hydrogen bonds between HPMC chains and the inorganic silicate network. For SGF-HPMC-SOL, this band becomes broader and its relative intensity decreases compared with SGF-HPMC. This change is consistent with a modification of the hydrogen-bonding environment. The broader O–H band indicates a more heterogeneous distribution of hydroxyl-related interactions, while the reduced relative intensity may be associated with stronger interfacial interactions among HPMC, silicate species, and silica sol particles.

The absorption band near 1440 cm^−1^ is mainly attributed to carbonate-related vibrations, which are associated with carbonate species generated from the reaction between sodium silicate and sodium bicarbonate [20]. The gradual decrease in the intensity of this band from SGF to SGF-HPMC and SGF-HPMC-SOL may reflect partial confinement or interaction of carbonate species within the composite network, as the organic–inorganic framework becomes denser. This variation is consistent with the formation of a more compact gel structure after the incorporation of HPMC and silica sol.

The band near 850 cm^−1^ can be assigned to Si–OH-related vibrations or symmetric vibrations associated with silicate species. In SGF-HPMC, the change in this band indicates that the introduction of HPMC affects the local environment of silanol groups through hydrogen bonding with hydroxyl and ether groups on the polymer chains. After the addition of silica sol, the relative intensity of the 850 cm^−1^ band decreases, suggesting a reduction in detectable free Si–OH environments or a change in the local bonding environment of silicate species. This result is consistent with enhanced silicate network development and stronger organic–inorganic interfacial interactions in the ternary system.

The strong band near 1100 cm^−1^ may originate from the stretching vibration of Si–O–Si related structures in the silicate network [21]. Compared with SGF, the SGF-HPMC-SOL sample exhibits a more intense and sharper band in this region, indicating the formation of a more integrated and silica-rich network. This spectral change is consistent with silica sol contributing to the reinforcement of the inorganic silicate framework, while HPMC provides polymer-chain entanglement and hydrogen-bonding sites that help connect the organic and inorganic phases. Therefore, the evolution of the 1100 cm^−1^ band is consistent with the formation of a strengthened organic–inorganic hybrid network.

Overall, the FTIR results reveal systematic changes in the O–H, carbonate-related, Si–OH-related, and Si–O–Si related bands with the sequential introduction of HPMC and silica sol. These changes indicate that HPMC mainly enhances hydrogen bonding and polymer-assisted network formation, whereas silica sol promotes silicate-rich network reinforcement and nanoscale filling [19,20,21]. Therefore, the FTIR results are interpreted as qualitative evidence supporting enhanced organic–inorganic interactions and the formation of a denser hybrid network. This structural evolution is consistent with the improved water retention, viscosity, thermal stability, and combustion resistance observed for SGF-HPMC-SOL.

### 3.8. Analysis of Silicon-Based Curing Foam in Combustion Experiments

**Figure 11:**
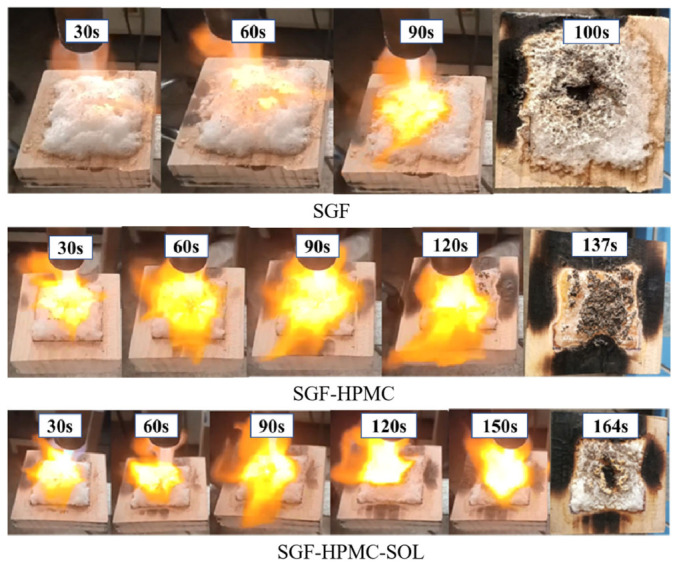
Image sequence of the burning process.

To intuitively evaluate the practical flame-retardant performance of the composite gel foam, its protective capability under sustained burning was tested, and morphological changes were recorded (as shown in Figure 11). The results indicate significant differences in the effective protection time among the systems, ranked as follows: ternary composite system (SGF-HPMC-SOL) > binary composite system (SGF-HPMC) > base system (SGF).

The combustion resistance test indicates the important role of silica sol incorporation in improving the flame-retardant performance of the gel foam. The enhanced performance can be mainly attributed to the reinforced organic–inorganic hybrid structure formed by HPMC, water glass, and silica sol. This structure helps maintain the integrity of the protective layer under flame exposure and delays direct heat transfer to the underlying wood substrate. In addition, the silica-containing components contribute to the formation of a silica-rich residue layer after combustion, which further acts as a physical thermal barrier. This test is a laboratory-scale screening assessment, not a full validation under real wildfire conditions. Therefore, the superior carbonization resistance of SGF-HPMC-SOL should be understood as the result of combined water retention, structural reinforcement, and silica-rich barrier effects.

A similar time-to-char approach has been adopted in recent studies on water-enhancing fire-protective gels. For example, Dong et al. exposed hydrogel-coated plywood substrates to a hand torch and used the time required for visible wood charring as a fire-protective metric; their test was described as a variation of the “Evaluation of Wildland Fire Chemicals Standard Test Procedures” adapted from ASTM E1321-1997(02) [5]. In that study, polymer–particle hydrogels protected wood substrates for more than 5–7 min, whereas the commercial water-enhancing gel AquaGel-K (ICL Performance Products LP, St. Charles, MO, USA) protected the substrates for less than 90 s [5].

### 3.9. Morphology Analysis of Silicon-Based Curing Foam After Combustion

**Figure 12:**
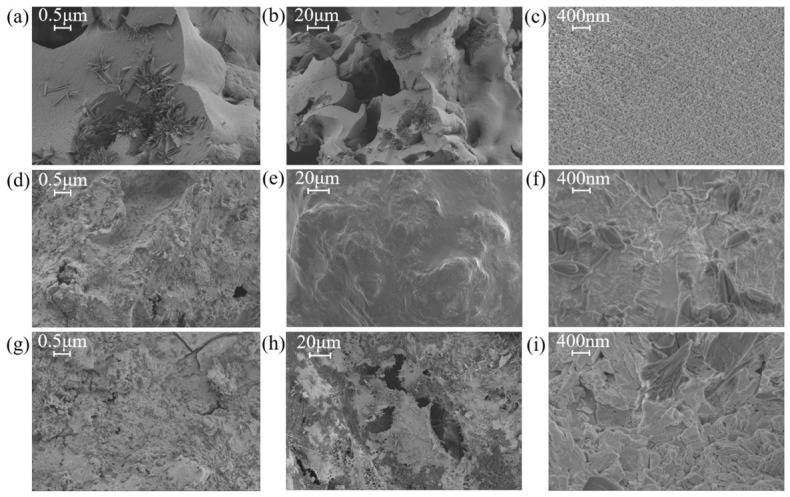
SEM microscopic morphology of different composite gels after combustion (**a**–**c**) SGF (**d**–**f**) SGF-HPMC (**g**–**i**) SGF-HPMC-SOL.

To investigate the mechanical behavior of the materials after combustion, the carbonized surfaces were observed using scanning electron microscopy (SEM). Figure 12 shows SEM microscopic morphology of different composite gels after combustion, where Figure 12a–c is SGF, Figure 12d–f is SGF-HPMC, and Figure 12g–i is SGF-HPMC-SOL. As shown in Figure 12b, the fracture surface of the pure water glass gel is flat and smooth, exhibiting typical brittle fracture characteristics. In contrast, the fracture surface of the water glass/HPMC/silica sol composite system (Figure 12h) is rough and uneven, showing features consistent with ductile-like deformation. For the water glass/HPMC binary system, the observed surface (Figure 12e) displays an intermediate flatness between the other two, indicative of intermediate toughness performance. Furthermore, crystalline substances were observed in the combustion residues of all systems (e.g., Figure 12a,f,i), and their specific phases will be analyzed subsequently by XRD. These morphological results indicate that the water glass/HPMC/silica sol composite system effectively improves the material’s toughness, enabling it to maintain better structural integrity even after combustion.

### 3.10. XRD Analysis of Silicon-Based Curing Foam After Combustion

**Figure 13:**
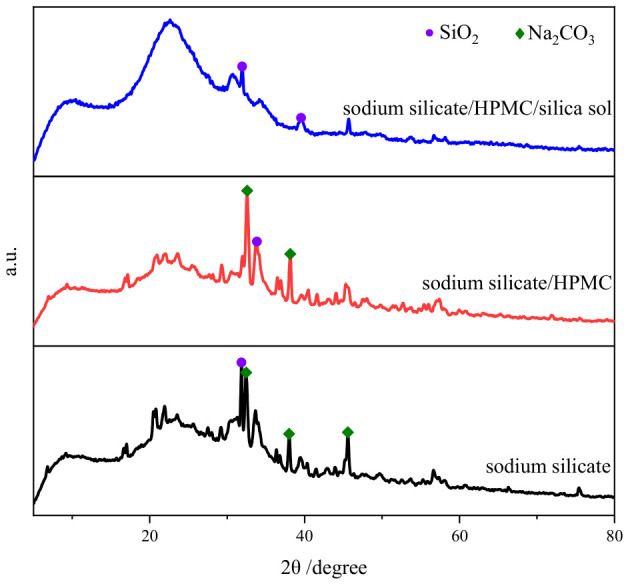
XRD patterns of different component gels.

To investigate the composition of the foam gel after the combustion test and the crystals observed on the surface in the aforementioned SEM images, XRD experiments were conducted. The three burned gel samples with different compositions are denoted as SGF, SGF-HPMC, and SGF-HPMC-SOL, SGF for water glass gel foam, SGF-HPMC for water glass/HPMC composite gel foam, and SGF-HPMC-SOL for water glass/HPMC/silica sol composite gel foam. The XRD patterns of different component gels are shown in Figure 13.

The results indicate that all three burned gel foams exhibit broad hump peaks at around 10° and 20°, which arise from amorphous silica generated during high-temperature decomposition of water glass and silica sol. SGF-HPMC-SOL shows the highest broad hump peak at 20°, consistent with the presence of silica-rich residue derived from the silica sol. Na_2_CO_3_ diffraction peaks are observed in SGF, indicating that the clustered crystals in SEM (Figure 12a) are Na_2_CO_3_ from NaHCO_3_ thermal decomposition. In SGF-HPMC and SGF-HPMC-SOL, clear SiO_2_ diffraction peaks appear, indicating that the layered crystals in SEM (Figure 12f,i) are SiO_2_.

### 3.11. Analysis of Forest Fire Barrier Test

**Figure 14:**
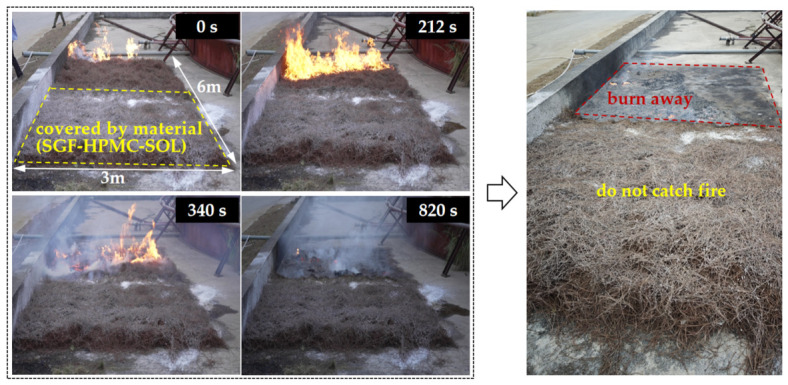
Fire spread process during one of the forest fire barrier tests.

To evaluate the performance of the material in a forest environment, two forest barrier tests were conducted. Figure 14 shows the fire spread process during one of the forest fire barrier tests. As shown, at the start of the test (0 s), the untreated pine needles are ignited from the side farthest from the treated area covered by the material (SGF-HPMC-SOL). Since the pine needles in the untreated area are highly flammable and are not treated with a fire retardant, the flames spread rapidly. At 212 s after ignition, the flames nearly reach the treatment area. At 340 s, when the flames reach the needles covered by SGF-HPMC-SOL, they are gradually extinguished. At 820 s, the embers in the untreated area continue to burn until they are nearly extinguished, while the pine needles sprayed with SGF-HPMC-SOL remain unignited. In the end, when the untreated pine needles burn away, the treated pine needles still do not catch fire. The repeat experiment yielded the same results as described above: the flame spread across the untreated pine needle area but failed to ignite the treated area where SGF-HPMC-SOL had been sprayed, indicating that the material (SGF-HPMC-SOL) provides effective barrier protection.

## 4. Conclusions

Based on a comprehensive evaluation of gelation time, water retention, foam stability, and combustion resistance, the optimal formulation of the composite gel was determined to consist of 3% sodium silicate and sodium bicarbonate, 0.8% HPMC, and 5% silica sol. The resulting ternary composite gel exhibits excellent water retention, adhesion, stackability, and flame-retardant durability. Combined macroscopic and microscopic characterizations indicate that its flame-retardant mechanism likely arises from a triple synergistic effect. The gel layer physically covers and isolates oxygen, water evaporation absorbs heat and lowers temperature, and HPMC and silica sol may contribute to the formation of a stable, thermally resistant char layer through possible interfacial interactions. This provides an effective strategy and a solid theoretical basis for the development of high-performance materials for forest fire prevention.

It should be noted that this study is limited to laboratory-scale tests. Future work should consider evaluating the aging, rain resistance, UV radiation resistance, toxicity, biodegradability, and effects on soil and vegetation of the composite gel to further validate its practical application.

## Data Availability

The original contributions presented in this study are included in the article. Further inquiries can be directed to the corresponding authors.
